# Relationship between Tai Chi and the mood states, self-esteem, and subjective well-being of middle-aged and older adults: a cross-sectional study from China

**DOI:** 10.3389/fpubh.2026.1686008

**Published:** 2026-01-28

**Authors:** Yan Li, Yanbin Hu

**Affiliations:** 1 College of Liberal Studies (Sports Work Department), Chongqing Industry Polytechnic University, Chongqing, China; 2 Department of Physical Education Teaching, Beijing University of Chinese Medicine, Beijing, China

**Keywords:** middle-aged and older adults, mood states, self-esteem, subjective well-being, Tai Chi

## Abstract

**Objective:**

This study examines the influence of Tai Chi on subjective well-being among middle-aged and older adults (aged 45 to 65 years), and reveals the chain mediating role of mood states and self-esteem between the two, providing effective strategies for improving the physical and mental health level of middle-aged and older adults.

**Method:**

The Physical Activity Rating Scale (PARS-3), Profile of Mood States (POMS), Self-esteem Scale (SES), and Subjective Well-being Scale (SWS) were used to conduct a questionnaire survey among 710 middle-aged and older adults in Chongqing, China, and the relationships among variables were analyzed using SPSS 26.0 and Mplus 8.3 software.

**Results:**

(1) Significant gender differences were observed in mood (*t* = −3.00, *p* < 0.05), self-esteem (*t* = 2.49, *p* < 0.01), and subjective well-being (*t* = 3.70, *p* < 0.001) among middle-aged and older adults. (2) Tai Chi exercise volume showed a significant negative correlation with mood states (*r* = −0.33, *p* < 0.001), while demonstrating significant positive correlations with self-esteem (*r* = 0.35, *p* < 0.001) and subjective well-being (*r* = 0.41, *p* < 0.001), respectively. (3) Tai Chi exercise volume directly and positively predicted subjective well-being in middle-aged and older adults (*β* = 0.47, *p* < 0.001). (4) Mood states (standardized effect size is 0.16) and self-esteem (standardized effect size is 0.04) each exerted partial mediating effects between Tai Chi exercise and subjective well-being. (5) The chained mediation pathway “mood states→self-esteem” demonstrated a statistically significant indirect effect (standardized effect size is 0.07) connecting Tai Chi exercise to subjective well-being.

**Conclusion:**

Maintaining sound mental health is imperative for middle-aged and older adults. As a green, safe, and healthy non-pharmaceutical measure, middle-aged and older adults people should actively and regularly participate in Tai Chi exercise, which is positively related to the improvement of mood states, self-esteem, and subjective well-being level.

## 1 Introduction

Population aging represents a global trend and has become one of the significant social issues facing the majority of nations worldwide in the 21st century (1). As the world’s second-largest economy with the largest population, China is experiencing an increasingly pronounced demographic shift toward aging. According to 2023 statistics, individuals aged 45 years and older now account for 38% of China’s population. Furthermore, the proportion of the population aged 60 years and older has risen from 14.9% in 2013 to 21.1% in 2023 (2). It is projected that by 2035, China’s population aged 60 and above will exceed 400 million, accounting for over 30 percent of the total population, marking the nation’s transition into a more severe phase of population aging (3). Undoubtedly, population aging will exacerbate a range of societal challenges, including increased individual health risks and heightened burdens on healthcare systems. As individuals advance in age, middle-aged and older adults not only experience declines in physical functioning but also commonly face mental health issues (4). Concurrently, studies indicate that per capita medical expenditures for middle-aged and older adults are 3 to 5 times higher than those for younger populations. Their out-of-pocket healthcare costs significantly exceed the population average, with older adults alone accounting for approximately 63% of total national healthcare expenditures (5). In 2022, China released the *State Council Notice on Issuing the “14th Five-Year Plan” for National Aging Undertakings and older adults Care Service System Development. This plan emphasizes the proactive response to population aging and advocates for the enhancement of older adults’ health status across physical, psychological, and social dimensions (6). Consequently, enhancing the physical and psychological health of the middle-aged and older population represents a critical evidence-based strategy to mitigate the challenges posed by population aging.

As a comprehensive indicator measuring quality of life and mental health, well-being serves as a complementary concept that enhances other economic and social welfare measures, and is gaining increasing scholarly attention (7). Subjective well-being (SWB) refers to an individual’s global evaluation of their quality of life based on their own standards. It serves as a comprehensive indicator reflecting both quality of life and mental health, encompassing three primary dimensions: life satisfaction, positive affect, and negative affect (8–10). Middle-aged and older adults, as a special group about to enter old age and those who have already entered old age, are in a particularly important period of happiness (11). A high level of subjective happiness can not only promote the long-term development of physical and mental health and self-improvement of middle-aged and older adults, but also help achieve the goal of healthy aging (12). Therefore, enhancing subjective well-being among middle-aged and older adults constitutes an essential evidence-based pathway toward achieving healthy aging.

### 1.1 The relationship between Tai Chi exercise and subjective well-being

In the field of exercise psychology, an increasing body of research has focused on the positive effect of regular physical activity or exercise on individuals’ subjective well-being (13, 14), an effect that is particularly pronounced among the older adults (15). For example, a meta-analysis demonstrated that regular participation in physical activity is an effective way to enhance subjective well-being among older adults (16). Engaging in physical exercise can enhance physical and mental health, boost self-confidence, and promote interpersonal interactions, thereby contributing to improved quality of life and greater well-being among the older adults (15, 17). Among these activities, Tai Chi-as a quintessential example of traditional Chinese aerobic exercises, is classified as a low-to-moderate intensity aerobic activity and a consciousness-oriented mind–body practice. It is characterized by moderate intensity, gentle movements, sustainable adherence, and minimal requirements for equipment or facilities (18). These attributes render it particularly suitable as a community- and home-based health rehabilitation modality, demonstrating positive effects on improving the psychological well-being of middle-aged and older adults (19, 20). For instance, to examine the effects of Tai Chi on well-being among middle-aged and older adults, a cross-sectional survey study (21) was conducted. Their findings revealed that Tai Chi practitioners reported higher levels of subjective well-being, with particularly pronounced benefits observed in adults over 60. Additionally, post-exercise outcomes demonstrated gender differences: female participants exhibited significantly greater well-being benefits compared to their male counterparts following Tai Chi exercise. Furthermore, similar findings have been documented in adolescent populations (22, 23) and adult cohorts (24). These studies consistently indicate that Tai Chi exercise exhibits a significant association with individual subjective well-being, positively facilitating its enhancement. Notably, a dose–response relationship has been observed between exercise intensity and subjective well-being outcomes. Based on this evidence, we propose hypothesis H1: Tai Chi exercise positively predicts subjective well-being in middle-aged and older adults.

### 1.2 The mediating role of mood states between Tai Chi and subjective well-being

Mood states refer to contagious yet subtle and sustained emotional conditions that reflect an individual’s internal affective adaptation to situational contexts. Maintaining positive mood states constitutes a critical indicator of psychological well-being (25, 26). Empirical evidence confirms significant correlations between physical exercise and mood regulation in middle-aged and older adults, with long-term exercise participation significantly enhancing positive mood while reducing negative affective experiences (27, 28). For instance, Jiang demonstrated that Tai Chi exercise effectively modulates practitioners’ mood states and maintains psychological equilibrium (29). A study of middle-aged and older women revealed statistically significant reductions (*p* < 0.05) in five negative mood dimensions after sustained Tai Chi training: Tension, Anger, Fatigue, Depression, and Confusion. Concurrently, significant elevations were observed in Vigor—a core positive mood dimension. These findings indicate that prolonged Tai Chi exercise promotes directional improvement toward favorable mood states (25). Concurrently, empirical studies confirm Tai Chi’s significant mood-enhancing effects in specific clinical populations, including aviation personnel (30) and individuals with substance use disorders (31). Longitudinal evidence indicates that regular Tai Chi exercise facilitates mood regulation and stabilization (32). Crucially, improvements in mood states correlate with elevated subjective well-being, where sustained positive affect or emotional homeostasis predicts higher life satisfaction (33). Furthermore, integrative mind–body regulation combined with routine exercise demonstrably alleviates depressive symptoms in community-dwelling older adults, reducing negative affect while enhancing positive mood states, thereby improving both subjective well-being and quality of life (34). However, the tripartite relationship among these constructs remains unexplored. Based on this theoretical foundation, we propose hypothesis H2: Mood plays a mediating role between Tai Chi practice and the subjective well-being in middle-aged and older adults.

### 1.3 The mediating role of self-esteem between Tai Chi and subjective well-being

Self-esteem is a form of self-evaluation and emotional experience. It refers to the experience of positive feelings toward the self-acquired by individuals through social practice, serving as a crucial psychological resource encompassing both competence and worth (35, 36). Concurrently, self-esteem represents a key indicator influencing the mental health of older adults (37). Within the domain of exercise psychology, physical activity demonstrates significant associations with individuals’ self-esteem levels. For instance, Han observed that older adults regular exercisers exhibited significantly higher scores in both physical self-esteem and global self-esteem compared to their sedentary counterparts (38). Subsequent research confirms that older adults engaging in sustained Tai Chi exercise typically possess elevated self-esteem alongside superior mental health status (39). Crucially, self-esteem is established as a robust predictor of subjective well-being (40, 41), maintaining a positive correlation with older adults’ well-being and demonstrating significant predictive validity (37, 42). Mechanistically, recent evidence reveals that physical exercise not only directly influences older adults subjective well-being and self-esteem, but also that self-esteem mediates the pathway between physical activity and subjective well-being (43). Parallel findings emerge in collegiate populations, where physical activity demonstrates significant correlations with both self-esteem and subjective well-being, with self-esteem mediating the relationship between physical exercise and well-being (44). This evidence collectively indicates that physical exercise not only directly influences subjective well-being but also confers indirect benefits through self-esteem enhancement. However, it remains unclear whether Tai Chi exercise yields comparable psychological benefits. Therefore, we propose hypothesis H3: Self-esteem plays a mediating role between Tai Chi exercise and the subjective well-being in middle-aged and older adults.

### 1.4 The chain mediating role of mood and self-esteem between Tai Chi and subjective well-being

Collectively, extant evidence indicates that individuals’ subjective well-being is differentially influenced by physical exercise, mood states, and self-esteem. Moreover, both mood states and self-esteem may function as independent mediators in the relationship between physical exercise and subjective well-being. However, the tripartite relationship among Tai Chi exercise, mood states, self-esteem, and subjective well-being in middle-aged and older adults remains inadequately understood. The research indicates that factors closely associated with individual subjective well-being can be categorized into external and internal factors (8). Internal factors primarily include emotional states (such as depression and loneliness) and personality traits (such as self-esteem and optimism) (45, 46). The “broaden-and-build” theory posits that positive emotions broaden the scope of attention and cognition, thereby helping to build lasting resources that promote well-being (47, 48). Based on this theory, the study has found that positive emotions can generate beneficial effects on health by transforming traits such as self-esteem into adaptive capacities and personal resources that individuals can utilize to improve health outcomes (49). Meanwhile, the study has found that individuals with higher self-esteem may experience greater well-being, with self-esteem serving as a mediator between mental health and subjective well-being in older adults (50). From the perspective of exercise psychology, older adults who engage in exercise (particularly morning exercise) generally exhibit lower stress levels, higher energy, and greater self-esteem (51). Moreover, physical exercise has been shown to effectively alleviate negative mood states such as tension, anger, fatigue, and depression in older adults while boosting their sense of vitality, and to some extent, enhancing participants’ self-esteem levels (52). However, this study did not thoroughly elucidate the intrinsic relationships among these variables. Moreover, a meta-analysis by (53) Gao revealed significant associations between self-esteem levels and psychological states: Individuals prone to negative affect (such as depression and anxiety) typically exhibit lower self-esteem, while those with pessimistic future orientation tend to possess diminished self-esteem. It is reasonable to hypothesize a positive relationship between mood and self-esteem levels, whereby individuals with favorable mood states may exhibit enhanced self-esteem and psychological well-being. However, it is currently unclear what the path relationship is among Tai Chi exercise, mood states, self-esteem, and subjective well-being in the middle-aged and older adults. This has a positive effect on enriching the theory and clinical practice of exercise psychology. Based on this, this study proposes hypothesis H4: Mood and self-esteem have a chain mediating effect between Tai Chi and the subjective well-being of middle-aged and older adults.

## 2 Participants and methods

### 2.1 Participants

This study employed a random sampling method to conduct a questionnaire survey among middle-aged and older adults (aged 45 to 65 years) in Chongqing Municipality. Using a random number method, random sampling was conducted across nine central urban districts in Chongqing, China, resulting in the selection of three districts (Beibei, Yubei, and Jiulongpo). Subsequently, random questionnaire surveys were administered to samples from these selected districts, following the principle of balanced sample size allocation during the survey process. The survey venues were primarily selected from residential communities and designated Tai Chi practice facilities (such as public squares and training institutions). During questionnaire administration, staff members thoroughly explained the completion requirements and item meanings to participants, ensuring their full comprehension of the questionnaire content. Paper questionnaires were administered and collected on-site, with participants required to spend a minimum of 10 min completing them. A total of 710 questionnaires were distributed, with 695 completed questionnaires returned. Following descriptive statistics, value conversion, and missing value imputation, 48 invalid responses (due to incomplete key information, partial completion, random/inconsistent responses, or missing data) were excluded. This resulted in 647 valid questionnaires, yielding an effective response rate of 93.1%. The sample comprised 243 men (37.6%), with a mean (±SD) age of 50.68 ± 5.95 years, height of 1.62 ± 0.58 m, and weight of 52.37 ± 8.49 kg, and 404 women (62.4%), with a mean (±SD) age of 52.15 ± 6.03 years, height of 1.53 ± 0.37 m, and weight of 50.55 ± 7.63 kg. The study protocol was approved following the Declaration of Helsinki, and written informed consent was obtained from all participants before their involvement (Table 1).

**Table 1:** Basic demographic characteristics of the participants (*n* = 647).

Variable	Category	N	Percentage
Sex	Male	243	37.56%
	Female	404	62.44%
Employment status	Retired	364	56.26%
	Employed	283	43.74%
Residence registration	Rural	287	44.36%
	Urban	360	55.64%
Education level	Primary school or below	134	20.71%
	Junior high school	149	23.03%
	High school / Technical secondary school	186	28.75%
	College / Bachelor’s degree	159	24.57%
	Master’s degree or above	19	2.94%

### 2.2 Measurement tools

#### 2.2.1 Physical activity rating scale (PARS-3)

The Chinese version of the Physical Activity Rating Scale (PARS-3) developed by Liang (54) was administered to assess participants’ Tai Chi exercise volume. This scale consists of three questions, namely exercise intensity, exercise duration, and exercise frequency. The Likert 5-level scoring method was adopted for evaluation, and the exercise intensity and frequency were, respectively, scored as 1 to 5 points from low to high, and the exercise time was, respectively, scored as 0 to 4 points from low to high. The calculation formula for the total exercise volume is exercise intensity × exercise time × exercise frequency. The higher the score, the greater the exercise volume. Total exercise scores ranged from 0 to 100 points, with established physical activity classification thresholds: Low exercise volume (0–19 points), moderate exercise volume (20–42 points), and high exercise volume (43–100 points). The internal consistency coefficient of this scale is 0.86, and the test–retest reliability is 0.82.

#### 2.2.2 Subjective well-being scale (SWS)

The revised General Well-Being Scale developed by Duan (55) was administered to assess participants’ subjective well-being levels. This scale consists of 18 items, covering six dimensions: Health Concern, Energy Level, Life Satisfaction, Positive Mood, Behavioral Control, and Stress Adaptation. The total scale score was calculated by summing all dimension scores, with higher composite scores indicating greater psychological well-being. In the current study, the Cronbach’s *α* coefficient for the full scale was 0.86. Measurement model validation results: x^2^/df = 1.64，RMSEA = 0.04, AGFI = 0.96, TLI = 0.99, CFI = 0.95, IFI = 0.98, GFI = 0.94. These indices collectively demonstrate excellent construct validity of the measurement scale.

#### 2.2.3 Self-esteem scale (SES)

The Self-Esteem Scale (SES) developed by Rosenberg (56) was administered to assess participants’ self-esteem level. This scale has been validated for effectively measuring self-esteem levels in Chinese older adult populations (57). The scale comprises 10 items, with positively worded items (1, 2, 4, 6, 7) scored directly and negatively worded items (3, 5, 8, 9, 10) reverse-scored. This scale adopts a 4-point Likert scoring, where 1 = strongly disagree and 4 = strongly agree. The total score range of the scale is between 0 and 40. The higher the score, the higher the individual’s self-esteem level. In this study, the Cronbach’s *α* coefficient of the total scale was 0.85. Measurement model verification results: x^2^/df = 2.03, RMSEA = 0.05, AGFI = 0.97, TLI = 0.99, CFI = 0.94, IFI = 0.96, GFI = 0.97. It indicates that the reliability and measurement validity of this scale are both at a good level.

#### 2.2.4 Profile of mood states (POMS)

The Chinese version of the Profile of Mood States (POMS) developed by Zhu (58) was administered to assess participants’ affective states. This instrument comprises 40 affective adjectives organized into seven subscales: five measuring negative affect dimensions and two assessing positive affect dimensions. This scale uses a 5-point Likert scoring system. The total score of mood states is calculated as follows: negative emotions (tension + anger + fatigue + depression + panic) - positive emotions (energy + self-esteem) + 100. The higher the total score of an individual, the worse their mood states; conversely, the lower the score, the better their mood states. In the current study, the Cronbach’s *α* coefficient for the full scale was 0.92, while reliability estimates for individual subscales ranged from 0.72 to 0.91. Measurement model validation results: x^2^/df = 1.98, RMSEA = 0.04, AGFI = 0.96, TLI = 0.98, CFI = 0.93, IFI = 0.97, GFI = 0.98. These indices collectively demonstrate excellent psychometric properties with robust reliability and construct validity.

### 2.3 Statistical methods

The data were processed and analyzed using SPSS 26.0 statistical software. Among them, Descriptive statistics, independent sample t-test, and one-way analysis of variance were mainly used to analyze the differential characteristics among variables. Pearson correlation analysis and linear regression analysis were conducted on the correlations among Tai Chi exercise, mood, self-esteem, and subjective well-being. Serial mediation analysis was conducted using SPSS 26.0 and Mplus 8.3 to examine the chained mediating effects of self-esteem and mood states in the relationship between Tai Chi exercise and subjective well-being. Bootstrap mediation analysis was employed to examine pathway mechanisms and estimate mediation effect sizes, with statistical significance set at *p* < 0.05.

## 3 Results

### 3.1 Common method bias assessment

This study adopted the questionnaire survey method. All questionnaire items were filled out by the participants themselves, so there may be common methodological bias problems in the measurement. To minimize the impact on the research results, this study adopted program-controlled methods, such as anonymous questionnaire measurement and standardized measurement, to control the entire measurement process accordingly. After the data collection was completed, the Harman single-factor test was used to test for the possible common method bias of all variables (59). Exploratory factor analysis with an unrotated solution extracted nine factors satisfying Kaiser’s criterion (eigenvalues>1.00). The primary factor accounted for 27.24% of total variance, substantially below the critical threshold of 40%. These results indicate no substantial common method bias in the current dataset.

### 3.2 Distribution of Tai Chi exercise volume

Table 2 shows the distribution of the number of people with different amounts of Tai Chi exercise, among which 226 people (34.93%) had low exercise volume, 319 people (49.30%) had moderate exercise volume, and 102 people (15.77%) had high exercise volume.

**Table 2:** Statistical table of Tai Chi exercise volume distribution (*n* = 647).

Level	Male		Female		All	
	*n*	Percentage of Total Participants	*n*	Percentage of Total Participants	*n*	Percentage of Total Participants
Low exercise volume	45	6.96%	181	27.98%	226	34.93%
Moderate exercise volume	135	20.87%	184	28.44%	319	49.30%
High exercise volume	63	9.74%	39	6.03%	102	15.77%

### 3.3 Gender differences in Tai Chi exercise, mood states, self-esteem, and subjective well-being

Treating gender as a categorical variable, we conducted independent samples t-tests to examine differences in self-esteem, mood states, subjective well-being, and Tai Chi exercise volume among middle-aged and older adults. The results revealed significant gender differences in self-esteem, mood states, and SWB. In contrast, no significant difference was observed in Tai Chi exercise volume. Specifically, the gender difference in self-esteem reached a significant level (*t* = 2.49, *p* < 0.01), and it was significantly lower in females than in males. The gender difference in mood reached a significant level (*t* = −3.00, *p* < 0.05), and women had significantly higher scores than men. The gender difference in subjective well-being reached a significant level (*t* = 3.70, *p* < 0.001), with females significantly lower than males (Table 3).

**Table 3:** Analysis of gender differences in each major variable (*n* = 647).

Variable	Male (*n* = 243)		Female (*n* = 404)		*t*
	*M*	*SD*	*M*	*SD*	
Mood states	93.68	21.09	98.90	21.94	−3.00**
Self-esteem	33.24	4.15	32.40	4.19	2.49**
Subjective well-being	98.69	13.90	94.49	14.08	3.70**
Tai Chi exercise volume	20.86	12.80	19.47	12.06	1.39

*p < 0.05; **p < 0.01; ***p < 0.001.

### 3.4 Correlation analysis of Tai Chi exercise, mood states, self-esteem, and subjective well-being

The correlation coefficient matrix indicates (Table 4) that (1) the amount of Tai Chi exercise is significantly negatively correlated with the mood of middle-aged and older adults people (*r* = −0.33, *p* < 0.001), while it is significantly positively correlated with self-esteem (*r* = 0.35, *p* < 0.001) and subjective well-being (*r* = 0.41, *p* < 0.001), respectively. (2) The mood of middle-aged and older adults people was significantly negatively correlated with self-esteem (*r* = −0.70, *p* < 0.001) and subjective well-being (*r* = −0.75, *p* < 0.001), respectively. (3) The self-esteem of middle-aged and older adults people was significantly positively correlated with subjective well-being (*r* = 0.71, *p* < 0.001). It can be seen from the results that there is a significant correlation among the research variables, providing a good foundation for the subsequent test of the mediating effect.

**Table 4:** Correlation coefficient of Tai Chi exercise, mood states, self-esteem, and subjective well-being.

Variable	M ± SD	Exercise volume	Mood states	Self-esteem	Subjective well-being
Exercise volume	20.00 ± 12.36	1.00	1.00	1.00	1.00
Mood states	96.94 ± 21.75	−0.33***			
Self-esteem	32.71 ± 4.19	0.35***	−0.70***		
Subjective well-being	96.06 ± 14.15	0.41***	−0.75***	0.71***	

*** indicates *p* < 0.001.

### 3.5 Analysis of mediating effect test

**Figure 1:**
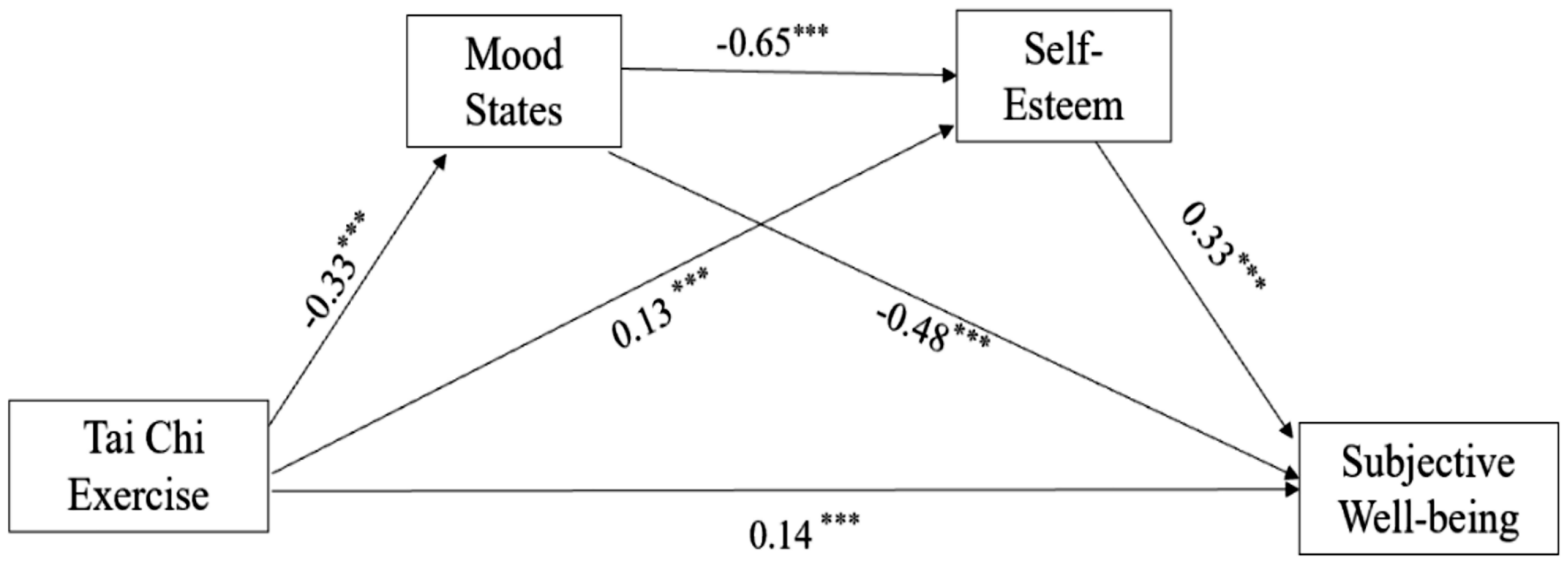
The chain mediating path diagram of mood states and self-esteem between Tai Chi and subjective well-being. ***p < 0.001.

To investigate the relationship among Tai Chi exercise, mood states, self-esteem, and subjective well-being, this study adopted the mediating effect test process proposed by Wen (60). Based on controlling for gender variables, 5,000 repeated samples were randomly conducted through the Bootstrap method to, respectively, test the direct effect of Tai Chi exercise on subjective well-being and the mediating effect of mood states and self-esteem between the two (Table 5). The results demonstrated that: (1) When examining the direct association using linear regression with Tai Chi exercise volume as the independent variable and subjective well-being as the dependent variable, exercise volume exhibited a significant direct positive predictive effect on well-being (*β* = 0.47, *p* < 0.001). (2) Regression analysis with exercise volume as the independent variable and mood states as the dependent variable revealed a significant direct negative predictive effect of exercise on mood (*β* = −0.33, *p* < 0.001). When exercise volume and mood states jointly predicted self-esteem in a multiple regression model, exercise volume demonstrated a significant direct positive predictive effect on self-esteem (*β* = 0.13, *p* < 0.001), while mood states showed a striking negative direct predictive effect (*β* = −0.65, *p* < 0.001). (4) When exercise volume, mood states, and self-esteem were simultaneously entered as predictors in a linear regression model with subjective well-being as the outcome variable, exercise volume demonstrated a significant direct positive predictive effect (*β* = 0.14, *p* < 0.001). Mood states significantly negatively predicted subjective well-being (*β* = −0.48, *p* < 0.001), while self-esteem exerted a significant positive predictive effect (*β* = 0.33, *p* < 0.001). Therefore, hypothesis H1 of this study has been confirmed (the mediation model diagram is shown in Figure 1).

**Table 5:** Regression analysis of mood states and self-esteem between Tai Chi and subjective well-being.

Variable	Exercise volume		Mood states		Self-esteem		Subjective well-being	
	*β*	*t*	*β*	*t*	*β*	*t*	*β*	*t*
Gender	−0.13	−3.74^***^	0.11	2.89^**^	−0.02	−0.70	−0.05	−2.28^*^
Exercise volume	0.47	11.01^***^	−0.33	−4.10^***^	0.13	4.43^***^	0.14	5.39^***^
Mood states					−0.65	−21.50^***^	−0.48	−14.06^***^
Self-esteem							0.33	9.75^***^
*F*	45.53^***^		27.03^***^		129.97^***^		203.10^***^	
*R* ^2^	0.22		0.10		0.50		0.66	
Δ*R*^2^	0.22		0.10		0.50		0.65	

*p < 0.05;**p < 0.01; ***p < 0.001. ΔR^2^ is the adjusted R^2^.

The test of the path relationship between variables revealed (Table 6) that in the mediation model, the total indirect effect was 0.27, accounting for 65.85% of the total effect. Within the serial mediation model, the specific indirect effect for the path “Tai Chi exercise→ Mood states →Subjective well-being” was significant (standardized effect size = 0.16, 95% CI excluding 0). This indicates that mood states function as a significant mediator in the relationship between Tai Chi exercise and subjective well-being, accounting for 39.02% of the total effect. Furthermore, the specific indirect effect through the path “Tai Chi exercise→Self-esteem -Subjective well-being” was statistically significant (standardized effect size = 0.04, 95% CI excluding 0). This indicates that self-esteem significantly mediated the relationship between Tai Chi exercise and subjective well-being, accounting for 9.76% of the total effect. The specific indirect effect through the chain mediation path “Tai Chi exercise→ Mood states → Self-esteem → Subjective well-being” was statistically significant (standardized effect size = 0.07, 95% CI excluding 0). This indicates that mood states and self-esteem sequentially mediated the relationship between Tai Chi exercise and subjective well-being, accounting for 17.07% of the total effect. Consequently, the proposed hypotheses (H2, H3, and H4) were fully supported.

**Table 6:** Standardized direct, indirect, and total effects of Tai Chi exercise on subjective well-being.

Effect type	Path	Effect size	Boot SE	Bootstrap95%CI		Proportion of Total effect (%)
				Lower	Upper	
Direct effect	Tai Chi →SWB	0.14	0.03	0.09	0.22	34.15
Indirect effects	Tai Chi →Mood states →SWB	0.16	0.02	0.14	0.23	39.02
	Tai Chi →Self-esteem →SWB	0.04	0.01	0.03	0.08	9.76
	Tai Chi →Mood states →Self-esteem →SWB	0.07	0.03	0.06	0.11	17.07
Total indirect		0.27	0.03	0.25	0.38	65.85
Total effect		0.41	0.04	0.39	0.55	100.00

## 4 Discussion

### 4.1 Gender differences in middle-aged and older adults’ mental health

This study revealed significant gender differences in mental health status among middle-aged and older adults. Specifically, women exhibited significantly higher mood state scores, whereas men demonstrated significantly greater self-esteem and subjective well-being. Collectively, these results indicate that male participants reported significantly more favorable mood states, coupled with higher levels of self-esteem and subjective well-being, relative to female participants. Previous research consistently indicates that middle-aged and older adult males tend to demonstrate superior mental health outcomes compared to females—a finding aligned with extant literature (61–63). Empirical evidence confirms these gender disparities across multiple domains, including heightened emotional resilience, enhanced cognitive performance, and greater adaptive functioning among males (61, 64). This observed gender disparity in mental health may stem from multifaceted factors, chief among them being women’s heightened emotional sensitivity and greater attentional focus on internal states and contextual details. These traits predispose women to amplified neuroaffective reactivity toward external stimuli (65). Furthermore, cultural dynamics specific to China may amplify these gender disparities. Traditional gender role socialization grants retired men greater psychosocial liberation, facilitating detachment from occupational obligations and reduced domestic burdens. Conversely, women typically retain disproportionate caregiving responsibilities for households and children, creating sustained role strain. This divergence in post-retirement role allocation contributes significantly to men’s superior mood states and elevated subjective well-being.

### 4.2 Direct relationship between Tai Chi and subjective well-being in middle-aged and older adults

The findings demonstrate a significant positive correlation between Tai Chi exercise volume and subjective well-being in middle-aged and older adults, with exercise volume exhibiting a direct positive predictive effect on well-being. This indicates that higher engagement in Tai Chi exercise predicts richer hedonic experiences within this population, aligning with prior research (21). As one of the measurement criteria for evaluating the level of individual life satisfaction, subjective well-being can effectively reflect the quality of life of an individual. With the development of exercise psychology, the improvement benefits of active physical exercise or sports participation on individual subjective well-being have been confirmed by researchers (66, 67). Researchers emphasize that physical exercise can not only increase an individual’s social activity but also improve social support and interpersonal relationship skills (68), and such relationships can bring positive well-being experiences to the participating individuals (17). Among them, Tai Chi exercise is an effective strategy to enhance an individual’s subjective well-being. For instance, studies have shown that college students’ positive emotions can be improved through regular participation in Tai Chi exercise. This not only effectively enhances college students’ positive emotions but also alleviates their negative emotions, thereby significantly improving their subjective well-being (69). Tai Chi exercise requires calming the mind and guiding the qi with the mind, emphasizing the unity of form and spirit. It has the effect of self-cultivation and moral improvement, and can effectively enhance the social skills and social participation of the older adults, as well as increase their subjective well-being, ultimately achieving the goal of improving physical and mental health (70). Overall, regular participation in Tai Chi exercise is closely associated with increased levels of subjective well-being in individuals. However, there are currently few studies focusing on the middle-aged and older adults group. This study provides cross-sectional research evidence for Tai Chi in improving the subjective well-being of the middle-aged and older adults. More sufficient direct evidence and the corresponding mechanism of action still need to be enriched in subsequent research.

### 4.3 The mediating role of mood states and self-esteem between Tai Chi and subjective well-being

To further elucidate the pathway relationship between Tai Chi exercise and subjective well-being in middle-aged and older adults, this study constructed a chain mediation model. The results revealed that mood states and self-esteem not only served as partial mediators independently in the relationship between Tai Chi exercise and subjective well-being, but also collectively formed a significant chain mediation pathway between Tai Chi and subjective well-being. Specifically, the identified serial mediation path was: “Tai Chi exercise→mood states → self-esteem → subjective well-being.” This indicates that the relationship between Tai Chi exercise and subjective well-being in middle-aged and older adults is substantially mediated by the serial intermediary roles of mood and self-esteem (with an effect of 0.27). Furthermore, a hierarchical and sequential relationship exists between mood and self-esteem, which collectively and partially explains the association between Tai Chi exercise and subjective well-being in this population (with an effect of 0.07).

On the one hand, Tai Chi exercise can indirectly predict subjective well-being in middle-aged and older adults through the mediating role of mood state. As evidenced by research, Tai Chi exercise demonstrates significant efficacy in maintaining emotional stability among older adults, particularly in alleviating negative affect such as depression and anger (71). This facilitates the regulation and maintenance of participants’ mood states (32). Simultaneously, compared to individuals with lower physical activity engagement, those who actively participate in physical exercise report higher levels of subjective well-being, it may be partially mediated through the influence of exercise on affective experiences (22). Research examining the benefits of integrated mind–body regulation combined with regular exercise has demonstrated that this approach significantly improves mood states in community-dwelling older adults with depression. Specifically, it enhances positive affect while reducing negative affect, consequently promoting greater subjective well-being (34). Therefore, as a mind–body exercise, Tai Chi demonstrates proven effectiveness in enhancing subjective well-being among middle-aged and older adults. This study further verifies that it can indirectly and positively predict subjective well-being through the mediating role of mood state. On the other hand, Tai Chi exercise can also indirectly and positively predict subjective well-being in middle-aged and older adults through the mediating role of self-esteem. For example, Chen (22) demonstrated that regular physical activity not only directly increases subjective well-being in adolescent populations but also exerts indirect effects through psychological variables such as self-esteem and personality traits. Consistent with these findings (72), similarly observed a significant positive correlation between physical activity and individuals’ subjective well-being, with self-esteem demonstrating a mediating role in this relationship. Based on the findings of this study, participation in Tai Chi enables middle-aged and older adults to fulfill interpersonal needs and generate positive emotions. These positive emotions are associated with enhanced self-esteem levels. Individuals with higher self-esteem tend to demonstrate more stable cognitive capacities when confronting dissatisfactory external environments, typically employing proactive and optimistic approaches to cope with setbacks and challenges, consequently exhibiting elevated subjective well-being.

More importantly, there exists a close relationship between individuals’ mood states and self-esteem, which plays a sequential mediating role in the connection between Tai Chi exercise and subjective well-being among middle-aged and older adults. Tai Chi exercise helps promote physical and mental relaxation, enhance positive emotions, and suppress negative emotions (73), while also fostering stronger self-esteem (51). According to the Broaden-and-Build Theory, positive emotions serve to broaden and build enduring resources that promote well-being (47, 48), and self-esteem appears to play a significant role in this process. Research has found that positive emotions are significantly positively correlated with self-esteem, whereas negative emotions demonstrate a significant negative correlation with self-esteem (74, 75). Joiner (76) established a significant positive association between negative affect and lower self-esteem, evidenced by comparatively poorer self-rated mood quality among individuals with diminished self-esteem relative to their high self-esteem counterparts. Under negative emotional conditions, individuals with low self-esteem are more prone to attending to negative information (77). In contrast, those with high self-esteem generally demonstrate greater life satisfaction and subjective well-being. Furthermore, self-esteem has been found to serve as a significant mediator between both positive/negative emotions and life satisfaction (78). This dynamic suggests that individuals experiencing optimal mood states demonstrate enhanced capacity for optimistic reappraisal when confronting complex challenges. Such adaptive processing facilitates positive affective valence, consequently reinforcing higher self-esteem. Conversely, compromised mood states predispose individuals to anxiety and depressive symptoms during adversity, triggering self-doubt regarding personal efficacy. Sustained negative affect perpetuates a downward spiral of self-concept erosion, progressively diminishing self-esteem. Consequently, this study delineates the relationship between mood states and self-esteem in middle-aged and older adults while establishing their sequential mediation in the Tai Chi exercise-subjective well-being pathway. It should be noted that the “predictive” effects derived from this cross-sectional study are statistical inferences, and their causal direction needs to be confirmed by future research.

### 4.4 Limitations

This study primarily examined the relationship between Tai Chi exercise and subjective well-being in middle-aged and older adults, and revealed the chain mediating role of mood states and self-esteem in this association. Nevertheless, certain methodological constraints warrant acknowledgement. For instance: (1) The cross-sectional design employed in this study relies exclusively on subjective self-report measures, inherently limiting causal inference. To mitigate potential biases associated with this methodology (such as common method variance), future research should incorporate longitudinal investigations to establish directional relationships among variables. (2) While this study established the serial mediation of mood states and self-esteem, the Tai Chi exercise-subjective well-being pathway likely involves additional mediating and/or moderating variables, warranting further empirical scrutiny. (3) The geographically restricted sampling frame (exclusively drawn from Chongqing, China) constrains population generalizability. Future investigations should incorporate multi-regional cohorts to examine sociocultural moderators within cross-regional comparative frameworks.

## 5 Conclusion

This study not only clarifies the gender differences in psychological levels among middle-aged and older adults (aged 45 to 65 years) but also reveals the pathway relationships between Tai Chi exercise and mood states, self-esteem, and subjective well-being. Specifically, Tai Chi exercise can not only directly predict the subjective well-being of middle-aged and older adults but also indirectly predict it through the chain mediating effects of mood states and self-esteem. This has positive implications for enriching the theory and clinical practice of exercise psychology. The results suggest that Tai Chi is a green, safe, and healthy non-pharmaceutical measure positively associated with the mental health promotion of middle-aged and older adults. It calls on community neighborhood committees and relevant functional departments to enhance support for Tai Chi exercises among this population, foster a favorable exercise environment, and thereby safeguard and improve their physical and mental health.

## Data Availability

The original contributions presented in the study are included in the article/supplementary material, further inquiries can be directed to the corresponding author.
